# Changes in full blood count parameters in leptospirosis: a prospective study

**DOI:** 10.1186/1755-7682-7-31

**Published:** 2014-06-21

**Authors:** Nipun Lakshitha De Silva, MJR Niloofa, Narmada Fernando, Lilani Karunanayake, Chaturaka Rodrigo, H Janaka De Silva, Sunil Premawansa, Shiroma M Handunnetti, Senaka Rajapakse

**Affiliations:** 1 Tropical Medicine Research Unit, Faculty of Medicine, University of Colombo, 25 Kynsey Road, Colombo 08, Sri Lanka; 2 Institute of Biochemistry, Molecular Biology and Biotechnology, University of Colombo, Colombo, Sri Lanka; 3 Medical Research Institute, Colombo, Sri Lanka; 4 Faculty of Medicine, University of Kelaniya, Kelaniya, Sri Lanka; 5 Faculty of Science, University of Colombo, Colombo, Sri Lanka

**Keywords:** Leptospirosis, Platelets, Neutrophils, Lymphocytes

## Abstract

**Background:**

Leptospirosis presents diagnostic challenges to clinicians, in settings where other acute febrile illness are prevalent. The patterns of serial changes in haematological parameters in leptospirosis has not been evaluated previously.

**Methods:**

Clinical and laboratory data were collected prospectively from patients with leptospirosis in two hospitals in Sri Lanka. Leptospirosis was diagnosed based on WHO clinical criteria with confirmation using Microscopic Agglutination Test titre > 400 or 4 fold rise between acute and convalescent samples. Full blood count parameters were analysed up to the 14^th^ day of illness.

**Results:**

Data from 201 patients with leptospirosis were available. Leukocyte counts and absolute neutrophil counts showed a decline over the first 5 days of illness, then rose until the end of the second week. On day 3 of fever, the majority (75%) had normal leukocyte counts, and by day 5, leukocytosis was seen only in 38.1%; leucopenia was an uncommon finding. Lymphopenia was seen in over half on day 5, declining to just under a quarter of patients by day 10. Platelets declined over the first 6 days and then gradually rose. Thrombocytopenia was seen in nearly three-fourths of patients by day 5. Haemoglobin and haematocrit levels declined over the course of illness. Total white cell and neutrophil counts were higher, and haemoglobin and haematorcrit were significantly lower, in patients with severe disease.

**Conclusions:**

Neither leukocytosis nor lymphopenia were prominent features, while thrombocytopenia was seen during the 3^rd^ to 5^th^ day of illness, with dropping haemoglobin levels. Neutrophilia and low haemoglobin levels appear to predict severe disease. These findings may be of use to clinicians in differentiating leptospirosis from other acute infections like dengue, and could help in predicting severe leptospirosis.

## Introduction

Leptospirosis presents diagnostic challenges to the clinicians in the presence of other acute febrile illnesses such as dengue and typhus. Laboratory confirmation is often delayed, and clinicians treat leptospirosis based on clinical suspicion. The patterns of simple laboratory investigations are of potential use to the clinician in differentiating febrile tropical diseases. Changes in full blood counts in leptospirosis have been examined in two studies, however the patterns of serial changes in these parameters have not been examined previously [1,2]. Higher WBC and neutrophil counts have been demonstrated in patients with severe disease [3]. We conducted this study to determine the patterns of serial changes in full blood counts of patients with leptospirosis, and their relationship to disease severity.

## Methods

We carried out a prospective hospital-based study. All patients admitted to medical wards of the National Hospital, Colombo and Base Hospital Homagama, Sri Lanka with clinically suspected leptospirosis (based on WHO clinical criteria) were screened during a period of one year beginning May 2012, and those with serologically confirmed leptospirosis (microscopic agglutination test [MAT] >400 or a four-fold rise in titre between acute and convalescent samples) [4] were enrolled. MAT is the standard test for laboratory confirmation in Sri Lanka. Clinical and laboratory data were recorded until the point of discharge, transfer or death. Patients fulfilling any of the following criteria were defined to have developed severe leptospirosis: renal insufficiency (urine output < 400 ml per day, creatinine > 133 μmol/L, urea > 25.5 mmol/L, dialysis); jaundice (Bilirubin > 51.3 μmol/L), ICU stay, prolonged hospital stay (>10 days), death (note: there is no standard classification of severity for leptospirosis, thus severity was based on the presence of organ dysfunction, indicators of morbidity, and mortality). We analyzed full blood count parameters from admission up to the first 14 days from the onset of febrile illness. Comparison between severity of illness and haematological parameters was performed using two sample t-test.

## Results

**Figure 1:**
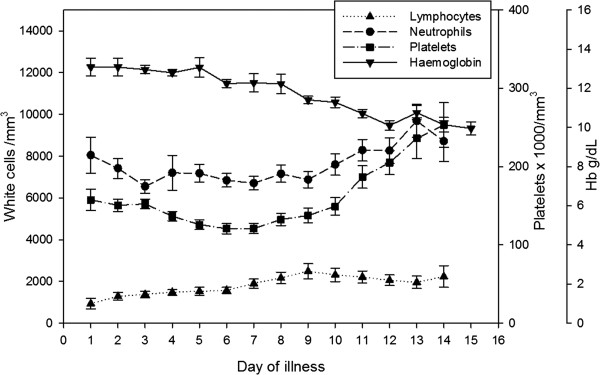
**Changes in haematological parameters over the course of illness.** Points indicate mean values, error bars represent standard error.

Of 539 patients screened, 201 had confirmed leptospirosis. Total white cell counts showed a decline over the first 5 days of illness, and then rose until the end of second week, with the highest mean (14643/μL) recorded on day 14; the absolute neutrophil count showed a similar pattern of change (Figure 1). On the 3^rd^ day of fever, the majority, i.e., 75%, had normal total white cell counts, with only a quarter of patients showing leukocytosis (>11000/μL). By day 5, high total white cell counts were seen in only 38.1% of patients, with the remaining 61.9% continuing to have normal white cell counts. Leukopenia (total white cell count <4000/μL) was seen in just 16% of patients on day 5 of illness. Lymphocyte counts were lower during the first week of the illness compared to the second. Lymphopenia (i.e., absolute lymphocyte count <1200) was seen in 54% on day 5, and in just 23% of patients by day 10.

**Figure 2:**
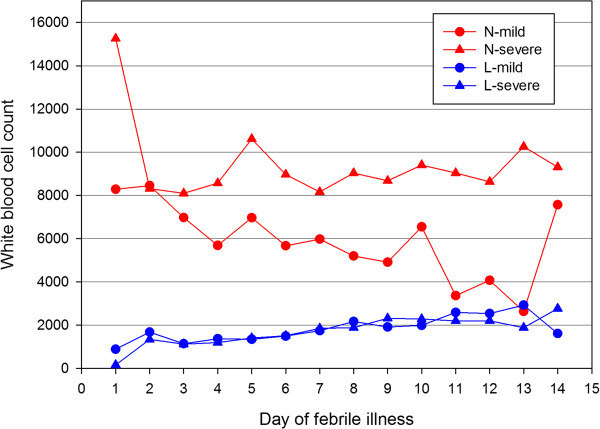
**Comparison of neutrophil and lymphocyte counts (/mm**^**3**^**) in mild and severe disease over the course of illness.** N-mild: neutrophil count in mild disease; N-severe: neutrophil count in severe disease; L-mild: lymphocyte count in mild disease; L-severe: lymphocyte count in severe disease.

**Figure 3:**
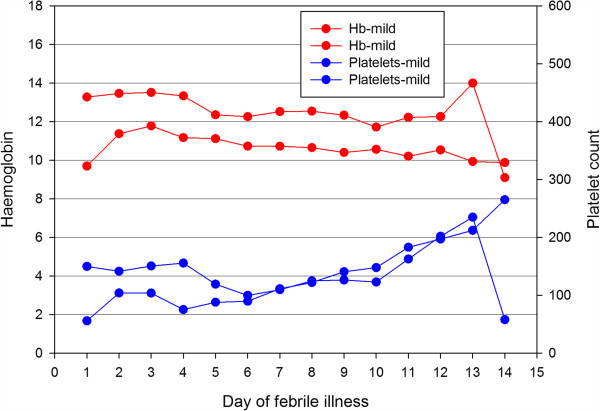
**Comparison of haemoglobin levels (g/dL) and platelet counts (/mm**^**3**^**) in mild and severe disease over the course of illness.** Hb-mild: haemoglobin in mild disease; Hb-severe: haemoglobin in severe disease; platelets-mild: platelet count in mild disease; platelet-severe: platelet count in severe disease.

Platelet counts showed a decline over the first six days of illness and then gradually rose. Thrombocytopenia (<150 × 10^9^/L) was seen in 56.76% of the patients on the third day, and this proportion rose to 73.8% by the fifth day. Thrombocytopaenia was seen in 80.7% at some point during the course of the illness. Haemoglobin levels showed a gradual decline over the course of illness.Mean haemoglobin and haematocrit levels were significantly lower in patients with severe leptospirosis compared to mild disease from day 3 to day 10 of illness (p < 0.001) (Figure 2). None of the patients required transfusion during this period. Platelets were significantly lower in severe leptospirosis compared to mild leptospirosis on day 3 and 4 of illness, but no significant differences were seen in platelet counts in the two categories from day 5 onwards. Total white cell and neutrophil counts were significantly higher in patients with severe leptospirosis from day 3 to day 8 of illness (p < 0.05) (Figure 3). Although lymphopaenia was seen in a proportion of patients, lymphocyte counts did not correlate with disease severity.

## Discussion

The findings described here are of potential use to clinicians in differentiating leptospirosis from viral infections such as dengue, and also bacterial sepsis. Normal or high leukocyte counts, lymphopenia, and lower haemoglobin and haematocrit values could potentially favour a diagnosis of leptospirosis over dengue. The changes in platelet counts are similar to patterns described earlier [1]. Our study supports the finding that thrombocytopaenia is not helpful in differentiating leptospirosis from dengue, as the pattern of reduction in platelet counts shown in this study shows similarities with dengue in the early stages (i.e., day 3 to day 5) of illness. Although neutrophil leukocytosis was not a prominent feature, leukocyte counts are normal throughout the course of illness in the majority; in contrast, nearly all patients develop leukopenia by the 3^rd^ day of illness in dengue [5]. Thus, the absence of leukopenia may differentiate leptospirosis from dengue. Lack of neutrophil leukocytosis may also help differentiate leptospirosis from bacterial sepsis.

In patients in whom a diagnosis of leptospirosis has been made clinically and serologically, the presence of neutrophilia and thrombocytopenia early on in the illness may predict the development of severe disease.

## Conclusions

Normal or slightly elevated leukocyte counts are seen in the majority of patients with leptospirosis; lymphopenia is not a characteristic feature, and platelets could be low. A neutrophilic response may predict severe disease. Lower haemoglobin and haematocrit levels on day 3–5 of illness may help differentiate leptospirosis from dengue.

## Ethics approval

Ethics clearance for the study was obtained from the Ethics Review Committees of the NHSL, and of the Faculty of Medicine, University of Colombo.

## Competing interest

The authors declare no conflict of interest.

## Authors’ contributions

SR, SMH, HJDeS and NLDeS planned the study. NLDS, TRGNF and MJRN collected data; NLDS and SR analyzed the relevant data and wrote the first draft. LK performed diagnostic tests for leptospirosis. All authors contributed towards critically reviewing and revising the draft, and approved the final manuscript. SR and NLDeS are guarantors of the paper.
